# Potential Interest in Circulating miR-BART17-5p As a Post-Treatment Biomarker for Prediction of Recurrence in Epstein-Barr Virus-Related Nasopharyngeal Carcinoma

**DOI:** 10.1371/journal.pone.0163609

**Published:** 2016-09-29

**Authors:** Nobuyuki Hirai, Naohiro Wakisaka, Satoru Kondo, Mitsuharu Aga, Makiko Moriyama-Kita, Takayoshi Ueno, Yosuke Nakanishi, Kazuhira Endo, Hisashi Sugimoto, Shigeyuki Murono, Hiroshi Sato, Tomokazu Yoshizaki

**Affiliations:** 1 Division of Otolaryngology-Head and Neck Surgery, Graduate School of Medical Science, Kanazawa University, Takara-machi 13–1, Kanazawa, 920–8640, Japan; 2 Division of Molecular Virology and Oncology, Cancer Research Institute, Kanazawa University, Kakuma-machi, Kanazawa, 920–1192, Japan; Gustave Roussy, FRANCE

## Abstract

**Objectives:**

Epstein-Barr virus (EBV)-related micoRNAs (miRNAs), BamHI-A rightward transcripts (BART)-miRNAs, are released in a stable form from viable cells, which are abundant in patients with EBV-positive nasopharyngeal carcinoma (NPC). We estimated copy numbers of circulating miR-BART2-5p, miR-BART17-5p, and miR-BART18-5p as well as BamHI-W DNA as biomarkers.

**Materials and Methods:**

Serums from 31 EBV-positive (confirmed by *in situ* hybridization for EBV-encoded small RNAs) NPC patients and 40 non-NPC controls were analyzed. Among the 31 NPC patients, serums at the initial diagnosis and three months after treatment were obtained from 20 patients, and serums only at three months after treatment were obtained from 11 patients.

**Results:**

The sensitivity/specificity of circulating BamHI-W DNA, miR-BART2-5p, miR-BART17-5p, and miR-BART18-5p for the diagnosis of NPC before treatment were 100 / 100, 85 / 85, 60 / 95, and 25 / 100%, respectively. For BamHI-W DNA, NPC patients with stage IV disease had significantly higher copy numbers than those with I-III. Copy numbers decreased significantly post-treatment. In contrast, copy numbers of the three BART-miRNAs showed no significant correlation with the clinical stage at diagnosis or any significant post-treatment change. After treatment, BamHI-W DNA and miR-BART17-5p were detected in 5 and 6 cases out of 11 patients with recurrent or residual tumors, respectively. However, BamHI-W DNA and miR-BART17-5p were absent in all 20 patients without relapse or residual tumors.

**Conclusion:**

The copy number of circulating BamHI-W DNA is a more useful biomarker for the initial diagnosis of NPC than the three BART-miRNAs examined. Post-treatment detection of miR-BART17-5p is a potential biomarker of a poor prognosis.

## Introduction

Nasopharyngeal carcinoma (NPC) is endemic in Southern China and Southeast Asia, but rare in most countries including Japan [1,2,3]. In addition to its geographic and population variations, NPC has some unique features. Etiologically, latent infection by Epstein-Barr virus (EBV) causes most cases of the non-keratinizing carcinoma differentiated type and non-keratinizing carcinoma undifferentiated type of NPCs, categorized as World Health Organization (WHO)-2A or WHO-2B NPC [4]. However, the contribution of EBV to the keratinizing squamous cell carcinoma, WHO-1, has been controversial. NPC generally shows the EBV-gene expression profile of type II latency; EBV-determined nuclear antigen-1, latent membrane protein (LMP)-1, LMP2, EBV-encoded small RNAs (EBERs), and BamHI-A rightward transcript (BART)-microRNAs (miRNAs) [5]. Clinically, NPC is highly invasive and metastatic, and, therefore, most NPC patients are diagnosed at an advanced stage [6,7]. Because of the anatomical features, the disease is not suitable for surgical resection [8]. Thus, radiotherapy plays a central role in the locoregional control of NPC [9,10]. Another characteristic of NPC is that metastasis to regional and/or distant sites is more common than for other carcinomas of the head and neck. Therefore, chemotherapy plays an important role in improving the treatment outcome [11].

We have been performing chemotherapy and radiotherapy alternately: first, chemotherapy, consisting of 5-FU (800 mg/m^2^/day on days 2–5) and cisplatin (100 mg/m^2^/day on day 6), is administered, and then a wide field of radiotherapy (36 Gy/ 20 fractions), chemotherapy, a shrinking field of radiotherapy (34 Gy/17 fractions), and chemotherapy [12]. Full-course treatment has been completed in 76.1% of patients, with overall and progression-free survival rates at 5 years of 78.04 and 68.74%, respectively. The data are at least comparable to those of the chemoradiotherapy arm of the American Intergroup Study 0099, which has been a mainstay in NPC treatment [11]. However, 30.1% of patients still develop recurrent or residual diseases. Therefore, there is still room to administer adjuvant chemotherapy (AC) following alternating chemoradiotherapy. It is inevitable that adding AC for all cases would increase treatment toxicity. Thus, biomarkers that can be used reliably to extract high-risk patients for recurrence and/or residual diseases are expected after treatment, so that we can solely perform AC for selected patients.

The consistent association between NPC and EBV-infection permits EBV-related factors to serve as biomarkers of NPC [13,14,15,16]. The EBV viral capsid antigen (VCA)/Immunoglobulin A (IgA) antibody titer has been used for NPC screening. However, the antibody titer is maintained at a high level after treatment in some NPC patients maintaining complete remission [14]. Therefore, the EBV VCA/IgA titer is not recommended as a reliable biomarker of the prognosis after treatment or for monitoring relapse. Now, quantitative analyses of copy numbers of circulating cell-free EBV-DNAs, especially BamHI-W DNA, by the quantitative polymerase chain reaction (qPCR) provide the most reliable biomarkers for NPC, the sensitivity and specificity of which are 93–96 and 92–100% for primary diagnosis, respectively [14]. The EBV-DNA loads at the initial diagnosis are correlated with the clinical stage and prognosis, and the detection of EBV-DNA after treatment was shown to be a predictive factor for recurrence and/or a residual tumor [14,17]. The source of cell-free EBV-DNA present in serum or plasma has been suggested to be derived from apoptotic NPC cells shed into the blood stream [14,15]. As the DNA loads are correlated with the clinical stage of NPC, estimated EBV-DNA copy numbers reflect the tumor volume at diagnosis and/or after treatment, but not the biological activity of viable tumor cells.

BART-miRNAs are non-coding miRNAs expressed abundantly in NPC cells [18]. EBV encodes 22 BART pre-miRNAs which can be further processed into more than 40 mature miRNAs [19]. BART-miRNAs are released in a stable form from viable cells enclosed in exosomes and/or independent of exosomes [20]. Moreover, several BART-miRNAs which are released into the blood are detected abundantly in NPC patients [21,22]. Thus, BART-miRNAs are new potential biomarkers for NPC to evaluate the biological activity of viable tumor cells. Although there have been some reports showing the clinical significance of miRNA at the initial diagnosis, no report has shown the significance of detecting circulating BART-miRNAs after treatment to predict the presence of residual tumor cells in NPC patients.

In the current study, we examined the copy numbers of several circulating BART-miRNAs as well as BamHI-W DNA before and after treatment, and evaluated their clinical utility as new potential biomarkers. We selected three of 44 mature BART-miRNAs, miR-BART2-5p, miR-BART17-5p, and miR-BART18-5p, which are already known to circulate abundantly in NPC patients [21].

## Materials and Methods

### Patient characteristics and serum collection

**Fig 1:**
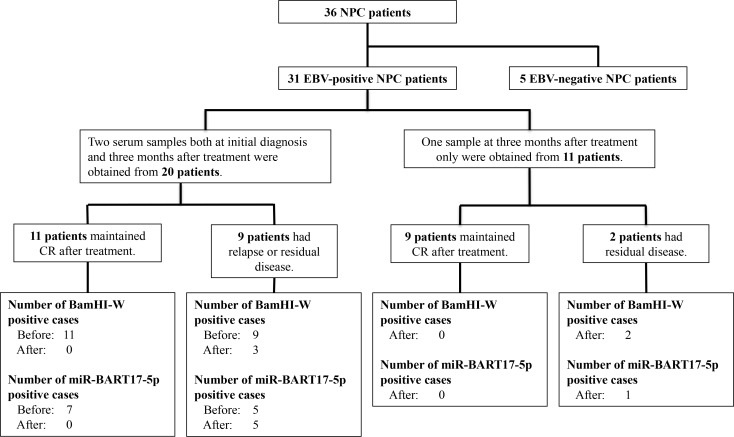
Flow chart of 31 NPC patients, with a summary of detection of the circulating BamHI-W DNA and miR-BART17-5p. 36 NPC patients were treated at Kanazawa University Hospital and Fukui-ken Saiseikai Hospital. Among them, thirty-one patients were confirmed as EBV-positive by *in situ* hybridization for EBERs (EBERs-ISH). Among the 31 NPC patients, serums at both the initial diagnosis and three months after treatment were obtained from 20 patients, and serums at three months after treatment only were obtained from 11 patients. After, 3 months after treatment; Before, before treatment (at the initial diagnosis); CR, complete remission; EBERs, EBV-encoded small RNAs; EBV, Epstein-Barr virus; and NPC, nasopharyngeal carcinoma.

This study was performed with the approval of the Kanazawa University Ethics Committee (2015–237). Blood samples were collected from 71 donors after providing written informed consent from August 2010 to December 2015. For serum collection, venous blood (9 mL) was collected and placed at room temperature for 1 hour, and then centrifuged at 1,600 g for 10 minutes at 4°C. The serum was collected gently, aliquoted, and frozen at -80°C until use. During the period, 36 NPC patients were treated at Kanazawa University Hospital and Fukui-ken Saiseikai Hospital. Among them, thirty-one patients were confirmed as EBV-positive by *in situ* hybridization for EBERs. Among the 31 NPC patients, serums at both the initial diagnosis and three months after treatment were obtained from 20 patients, and serums at three months after treatment only were obtained from 11 patients (Fig 1). The characteristics of the thirty-one patients are summarized in Table 1. The characteristics in Table 1 are shown separately for 20 patients with serum at both the initial diagnosis and three months after treatment, and for 11 patients with serums at three months after treatment only. All patients had histologically proven NPC with clinical stage I to IVC disease using the staging system of the Union Internationale Contre le Cancer [23]. All these patients had evidence of active disease, thirty as the first occurrences and one as a recurrence. Twenty-seven of the 31 NPC patients were treated with alternating chemoradiotherapy, as described in Introduction, and in detail elsewhere [12]. One patient was treated with endoscopic nasopharyngectomy for local relapse of the disease, which had been treated at another institution [8]. Three patients (two T1N0M0, and one T4N2M1) were treated with radiotherapy alone.

**Table 1:** Clinical characteristics of patients with nasopharyngeal carcinoma.

	Number of patients		
Characteristics	Patients with serum at both initial diagnosis and after treatment	Patients with serum after treatment only	Summary of all patients
Total	20	11	31
Sex			
male	19	8	27
female	1	3	4
Age			
range	16–68	44–81	16–81
mean	56.3	60.1	57.6
Tumor status			
T1	7	4	11
T2	3	1	4
T3	6	2	8
T4	4	4	8
Nodal status			
N0	4	3	7
N1	5	2	7
N2	7	5	12
N3	4	1	5
Metastasis status			
M0	18	9	27
M1	2	2	4
Clinical stage			
I	1	1	2
II	2	1	3
III	9	4	13
IVA	4	3	7
IVB	2	0	2
IVC	2	2	4
Histology (WHO Type)			
Keratinizing squamous cell carcinoma	0	0	0
Non-keratinizing carcinoma Differentiated type	15	7	22
Non-keratinizing carcinoma Undifferentiated type	5	4	9
Treatment			
Alternating CRT	18	9	27
Radiotherapy alone	1	2	3
Surgery	1	0	1

CR, complete remission; CRT, chemoradiotherapy; and WHO, World Health Organization. TNM stage classification was based on the staging system of the Union Internationale Contre Le Cancer [23].

Blood samples were collected at the initial diagnosis and/or three months after treatment for NPC patients. Again, among 31 NPC patients, serums at both the initial diagnosis and three months after treatment were obtained from 20 patients, and serums only at three months after treatment were obtained from 11 patients. Seven of the thirty-one patients had relapsed, and four of the thirty-one had a tumor-bearing state at a distant site after treatment. Twenty of the thirty-one patients maintained complete remission after treatment (Fig 1).

Control samples were obtained from 20 patients admitted to Kanazawa University with non-NPC head and neck carcinomas (5 oral carcinomas, 5 oropharyngeal carcinomas, 5 hypopharyngeal carcinomas, and 5 laryngeal carcinomas) at diagnosis, and from 20 healthy volunteers.

### Cell lines, and extractions of DNA and RNA from cells and culture media

Jijoye, a latency type III EBV-positive Burkitt’s lymphoma cell line, and BL2, an EBV-negative Burkitt’s lymphoma cell line, which were purchased from Deutsche Sammlung von Mikroorganismen und Zellkulturen (Braunschweig, Germany), were cultured in RPMI-1640 medium (Thermo Fisher Scientific, Tokyo, Japan) supplemented with 10% fetal bovine serum (Thermo Fisher Scientific, Tokyo, Japan), and 1% Antibiotic-Antimycotic Mixed stock solution (Nacalai Tesque, Kyoto, Japan), and were incubated in 5% CO_2_ at 37°C.

Total DNA and RNA were extracted from Jijoye and BL2 cells, and from each 10 mL of cultured media, respectively. Total DNA and RNA were extracted using the QIAamp DNA Mini kit (Qiagen, Tokyo, Japan) and RNeasy Plus mini Kit (Qiagen, Tokyo, Japan), according to the manufacturer’s protocol, respectively.

### Extraction of nucleic acids from serum samples

Total nucleic acid was extracted from a 500-μL aliquot of serum by the QIAamp Circulating Nucleic Acid kit (Qiagen, Tokyo, Japan) following the manufacturer’s protocol. During the process of nucleic acid extraction, we added synthetic *C*. *elegans* microRNA, cel-miR-39 (Hokkaido System Science, Sapporo, Japan), the so-called spike-in control. Cel-miR-39, which shows no sequence homology with any human microRNA, was used as an internal control for variations during the preparation and recovery of RNA, cDNA synthesis, and real-time qPCR. Due to a lack of generally accepted standards, all experimental real-time qPCR data for BART-miRNAs were normalized to cel-miR-39. Finally, it was eluted in 60 μL of DNase/RNase-free water and stored at -80°C until use. The recovery of RNA extracted from serum samples was assessed using Bioanalyzer (Agilent Technology, Tokyo, Japan), but the assay did not have enough sensitivity. Aliquots of nucleic acid for miRNA analyses were treated with RNase-Free DNase Set (Qiagen, Tokyo, Japan) following the manufacturer’s protocol before use.

### Construction of a plasmid

We generated a plasmid with 94 bp of BamHI-W DNA inserted. The BamHI-W DNA was amplified from extracted DNA of an EBV-positive epithelial cell line, NPC-KT cells, by PCR amplification using KOD FX Neo DNA polymerase (TOYOBO, Osaka, Japan). The primers used for plasmid construction were: 5’-CTGCTAAGCCCAACACTCC-3’ (forward), and 5’-ACCGGTGCCTTCTTAGGAGC-3’ (reverse). The fragment was inserted into the pTA2 vector (TOYOBO, Osaka, Japan).

### Reverse transcription

The synthetic miRNA mimics based on the TaqMan MicroRNA Assays list (Applied Biosystems, Tokyo, Japan) were purchased from Hokkaido System Science (Sapporo, Japan). Reverse transcription (RT) reactions were performed using miRNA-specific looped RT primers of TaqMan MicroRNA assays (Applied Biosystems, Tokyo, Japan) and the TaqMan MicroRNA RT Kit (Applied Biosystems, Tokyo, Japan), according to the Manufacturer’s protocol.

### Absolute quantification of BamHI-W DNA-fragment and BART-miRNAs by real-time qPCR

All qPCR assays were performed with MX3000P (Agilent Technology Stratagene, Tokyo, Japan). All tests were duplicated, and all cycle threshold (Ct) values were determined with standard curves in each qPCR. The cut-off Ct values were determined at 40 cycles.

Primers and a probe used to evaluate the copy numbers of BamHI-W DNA-fragment were: 5’-CTGCTAAGCCCAACACTCC-3’ (forward), 5’-ACCGGTGCCTTCTTAGGAGC-3’ (reverse), and 5’-(FAM)CACACACTACACACACCCACCCGTCTC(TAMRA)-3’ (probe). Real-time qPCR was performed using Thunderbird Probe qPCR Mix (TOYOBO, Osaka, Japan). A total of 100-fold serial dilutions of the plasmid described above and water as a negative control were used to generate a standard curve to estimate the absolute copy numbers. The qPCR cycle conditions were: 95°C for 1 min, then 40 cycles at 95°C for 15 sec, and 60°C for 1 min. According to the protocol of Lin et al., samples with an undetectable EBV signal after processing real-time qPCR conditions (40 cycles) were considered to contain zero copies [16].

Each miRNA load was determined by the qPCR assay using the primer sets of TaqMan MicroRNA Assays (Applied Biosystems, Tokyo, Japan). Real-time qPCR was performed using TaqMan Universal PCR Master Mix II w/o UNG (Applied Biosystems, Tokyo, Japan). A total of 100-fold serial dilutions using cDNA from each synthetic miRNA-mimic by RT reactions, as described above, and water as a negative control were used to generate each standard curve of miRNAs to estimate the absolute copy numbers. RT reactions and qPCR assays of the miRNA mimics were performed in parallel with tested samples. The qPCR cycle conditions were: 95°C for 10 min, then 40 cycles at 95°C for 15 sec, and 60°C for 1 min. We calculated the recovery rate based on cel-miR-39 as an exogenous control, and corrected copy numbers of each BART-miRNA based on this recovery rate. The corrected copy numbers served as each absolute quantity. As well as the BamHI-W DNA-fragment, samples were considered detectable if the amplification signal occurred before the 40^th^ threshold cycle.

### Statistical analyses

The associations of copy numbers of EBV-related DNA and miRNAs with clinical stages were tested using the Mann-Whitney *U* test. The Wilcoxon signed rank test was used to test the significance of differences between copy numbers before and after treatment. The differences in copy numbers between NPC patients and controls were assessed by the Mann-Whitney *U* test. We used SPSS statistics 23 software for statistical analyses, and the significance was defined as a *p*-value less than 0.05.

## Results

### Detection of BamHI-W DNA and BART-miRNAs in EBV-positive and EBV-negative cell lines

**Fig 2:**
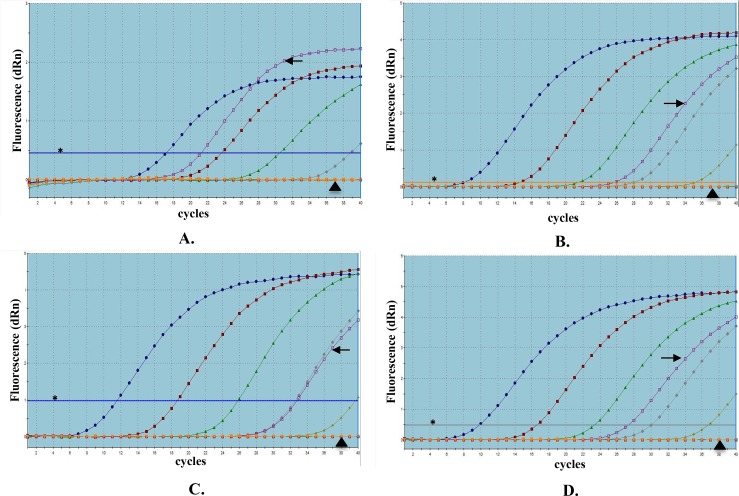
Amplification plots of BamHI-W DNA and three BART-miRNAs extracted from cell lines, by real-time qPCR. (A) BamHI-W DNA; (B) miR-BART2-5p; (C) miR-BART17-5p; and (D) miR-BART18-5p. Jijoye is a latency type III Epstein-Barr virus-positive Burkitt’s lymphoma cell line, and BL2 is an EBV-negative Burkitt’s lymphoma cell line. Standard curves are expressed with blue, red, green, grey, and yellow lines using 100-fold serial dilutions of plasmid or cDNA derived from BART-miRNA mimics. Each amplication plot of Jijoye and BL2 is expressed with purple and orange lines. A horizontal line indicated with an asterisk (✱) shows the threshold fluorescence based on each standard curve. Amplification plots of Jijoye and BL2 cell lines are indicated with an arrow (➜) and arrowhead (▲), respectively. Each EBV-related factor was detected only in the positive control, Jijoye, and not in the negative control, BL2.

We detected the BamHI-W DNA and three BART-miRNAs in Jijoye cells, a positive control, but not in BL2 cells, a negative control (Fig 2).

These results suggest that our system could detect these EBV-related DNA and miRNAs specifically.

### Estimating copy numbers of circulating BamHI-W DNA and three BART-miRNAs in NPC patients at diagnosis and non-NPC controls

**Fig 3:**
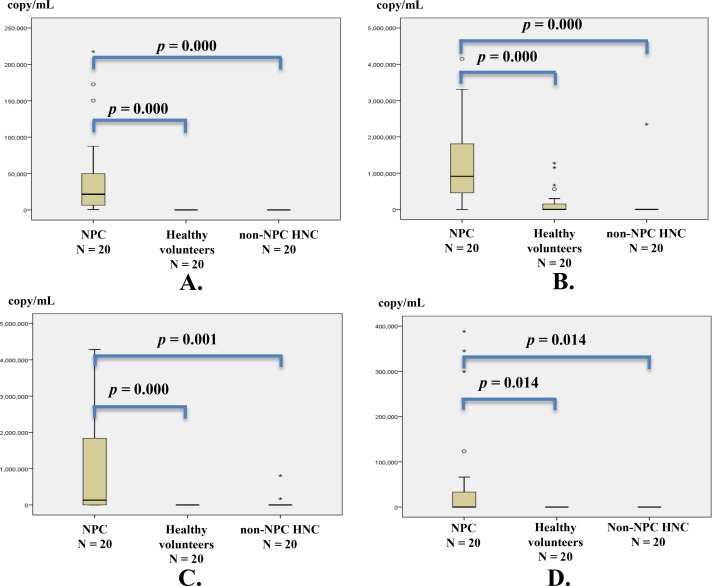
Copy numbers of circulating BamHI-W DNA and three BART-miRNAs in NPC patients at diagnosis and in non-NPC controls. (A) BamHI-W DNA; (B) miR-BART2-5p; (C) miR-BART17-5p; and (D) miR-BART18-5p. Serum samples from 20 NPC patients before treatment and from 40 non-NPC controls (20 non-NPC head and neck carcinomas, and 20 healthy volunteers) were analyzed. The copy numbers of BamHI-W DNA and the three BART-miRNAs were determined by qPCR and RT-qPCR, respectively. The copy numbers of BamHI-W DNA and the three BART-miRNAs in NPC patients at diagnosis were significantly higher than those in non-NPC controls. The copy numbers of BART-miRNAs were corrected by the recovery rate of cel-miR-39, an exogenous spike-in control. The significance of differences was assessed by the Mann-Whitney *U* test. The significance was defined as a *p*-value of less than 0.05. Circles (○) and asterisks (✱) show the statistical outliers, among which asterisks are extreme values. NPC, nasopharyngeal carcinoma; and HNC, head and neck carcinoma.

Copy numbers of circulating BamHI-W DNA and the three BART-miRNAs for each NPC patient before and after treatment are summarized in S1 Table. The BamHI-W DNA was detected in 20 of 20 NPC patients at diagnosis. The miR-BART2-5p, miR-BART17-5p, and miR-BART18-5p were detected in 17, 12, and 5 of the 20 NPC patients, respectively. The detection of circulating BamHI-W DNA and miR-BART17-5p at the initial diagnosis in 20 NPC patients are shown in flow chart (Fig 1). The copy numbers of circulating BamHI-W DNA and the three BART-miRNAs in NPC patients at the initial diagnosis were significantly higher than those in non-NPC controls (Fig 3). The circulating BamHI-W DNA was not detected in any non-NPC controls. However, the circulating miR-BART2-5p, miR-BART17-5p, and miR-BART18-5p were detected in 6, 2, and 0 out of 40 patients in the non-NPC controls (20 non-NPC head and neck carcinomas, and 20 healthy volunteers), respectively (S2 Table).

Each sensitivity/specificity of BamHI-W DNA, miR-BART2-5p, miR-BART17-5p, and miR-BART18-5p for the diagnosis of NPC before treatment were 100 / 100, 85 / 85, 60 / 95, and 25 / 100%, respectively.

**Fig 4:**
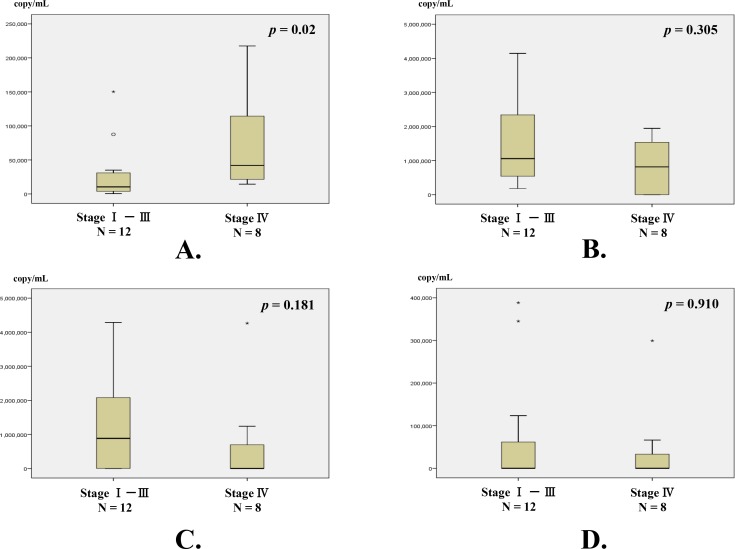
Correlation between copy numbers of circulating BamHI-W DNA and three BART-miRNAs, and clinical stage. (A) BamHI-W DNA; (B) miR-BART2-5p; (C) miR-BART17-5p; and (D) miR-BART18-5p. Classification of clinical stage from stage I to IVC was based on the staging system of the Union Internationale Contre le Cancer [23]. Twelve NPC patients had stage I-III disease, and 8 had stage IV. The copy numbers of BamHI-W DNA were significantly higher in patients with stage IV disease than in those with stage I-III disease. None of the three BART-miRNA were correlated with the clinical stage at the initial diagnosis. BART-miRNAs copy numbers were corrected by the recovery rate of cel-miR-39 as an exogenous spike-in control. The significance of differences was assessed by the Mann-Whitney *U* test. The significance was defined as a *p*-value less than 0.05. Circles (○) and asterisks (✱) show the statistical outliers, among which asterisks are extreme values.

Additionally, for the copy numbers of circulating BamHI-W DNA, there was a significant difference between stages IV and I-III at diagnosis (Fig 4). In contrast, the copy numbers of the three BART-miRNAs did not show any significant correlation with the clinical stage at diagnosis.

### Copy numbers of circulating BamHI-W DNA and three BART-miRNAs at diagnosis and their changes after treatment

**Fig 5:**
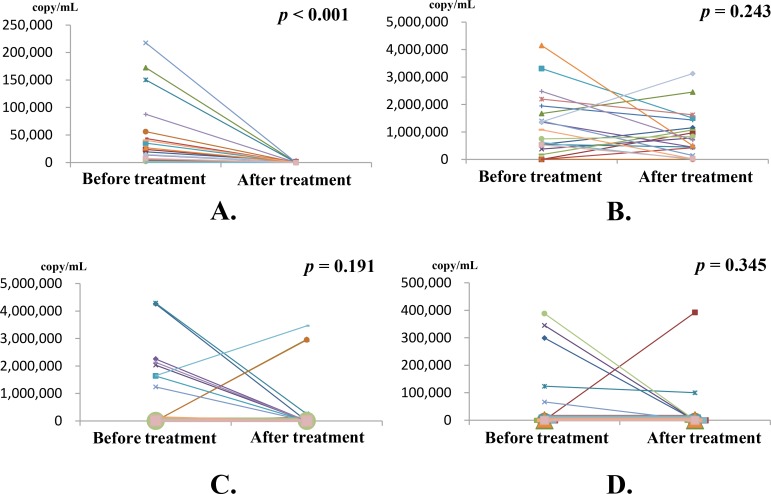
Copy numbers of circulating BamHI-W DNA and three BART-miRNAs at diagnosis and changes after treatment. The copy numbers of circulating BamHI-W DNA and the three BART-miRNAs were analyzed in serums from 20 patients with NPC before and three months after treatment. (A) The copy numbers of BamHI-W DNA before treatment were significantly decreased three months after treatment. (B) miR-BART2-5p; (C) miR-BART17-5p; and (D) miR-BART18-5p. No significant change in the copy number of any BART-miRNA was found. The change in copy number before and after treatment was linked in a line for each patient. BART-miRNA copy numbers were corrected by the recovery rate of cel-miR-39 as an exogenous spike-in control. The significance of differences was assessed by the Wilcoxon test. The significance was defined as a *p*-value less than 0.05.

Again, the copy numbers of circulating BamHI-W DNA and the three BART-miRNAs for each NPC patient before and after treatment are summarized in S1 Table. These changes in copy numbers of BamHI-W DNA and the three BART-miRNAs before and after treatment were analyzed in 20 NPC patients whose serums were obtained at both the initial diagnosis and three months after treatment. The copy number of BamHI-W DNA significantly decreased after treatment (*p* < 0.001) (Fig 5A). No BART-miRNA showed a significant change in copy numbers after treatment (Fig 5B–5D).

### Detection of circulating BamHI-W DNA and BART-miRNAs after treatment and its association with prognosis in 31 NPC patients

A summary of the detection of circulating BamHI-W DNA and BART-miRNAs after treatment and its association with the prognosis in 31 NPC patients are shown in S3 Table. Among 11 patients with relapse or residual disease, the circulating BamHI-W DNA-fragment was detectable in 5 patients. Two relapsed later, and three had distant metastasis before and after treatment. In contrast, no circulating BamHI-W DNA-fragment was detected in the 20 patients maintaining remission. The miR-BART17-5p was detected in 6 out of 11 patients with relapse or residual disease after treatment; 3 of them were newly detected cases after treatment. Four of the 6 patients relapsed later, and two were patients who had consistently shown distant metastasis since the initial diagnosis. Thus, these 6 miR-BART17-5p-detectable NPC patients after treatment all showed a tumor-bearing state. No circulating miR-BART17-5p was detected in any of the 20 patients maintaining remission. The detection of circulating BamHI-W DNA and miR-BART17-5p after treatment and its association with the prognosis of 31 NPC patients are summarized in Fig 1. The miR-BART18-5p was detected in only 2 of the 31 NPC patients after treatment. The 2 patients with elevated miR-BART18-5p loads after treatment developed distant metastases. The miR-BART 2-5p was still detected in 25 of the 31 NPC patients after treatment.

## Discussion

In the current study, the detection of serum BamHI-W DNA yielded both 100% sensitivity and specificity for the initial diagnosis of patients with NPC, which are comparable to past reports [14]. The sensitivity and specificity of miR-BART2-5p, miR-BART17-5p, and miR-BART18-5p were limited to 85 / 85, 60 / 95, and 25 / 100% for NPC at diagnosis, respectively. Thus, quantified copy numbers of the EBV-DNA provided a more useful biomarker for the diagnosis of NPC before treatment than the BART-miRNAs examined. The copy numbers of the DNA fragment were positively associated with the clinical stage of the disease at diagnosis. These results suggest that the serum EBV-DNA load detected by qPCR was correlated with the tumor volume. Actually, copy numbers of BamHI-W DNA in serum were markedly decreased after treatment. These data are compatible with previous reports showing the insufficient sensitivity of the EBV-DNA load in stage I NPC (50–86%) [14]. Although the origin of the circulating EBV-DNA detected by qPCR has been reported to be that derived from tumor apoptosis or necrosis shed into the blood stream [14], circulating viral RNAs would be derived from viable tumor cells *via* active extracellular transport. The copy numbers of these BART-miRNAs examined in the current study were not significantly correlated with the clinical stage at diagnosis. In addition, no significant changes in the copy numbers of any circulating BART-miRNAs were found after treatment. These data show that copy numbers of these circulating BART-miRNAs are affected by the activity in the production and/or secretion of these miRNAs from viable tumor cells rather than the tumor volume as BamHI-W DNA. Thus, the circulating BART-miRNAs may be biomarkers playing important roles in monitoring these activities of viable tumor cells regardless of the tumor volume.

The circulating miR-BART2-5p was still detected in 25 of the 31 NPC patients at 3 months after treatment. The circulating miR-BART18-5p was detected in only 2 of the 11 patients with relapse or residual disease. These data show that miR-BART2-5p and miR-BART18-5p are not favorable candidate biomarkers with high sensitivity and/or specificity after treatment to make a prognosis. At 3 months after treatment, the BamHI-W DNA was detected in 5 of the 11 NPC patients with relapse or residual diseases, and the EBV-DNA was not detected in the 20 cases with remission. The circulating miR-BART17-5p was detected in 6 of the 11 patients with relapse or residual tumors, whereas the miRNA was not detected in any of the 20 patients with remission. These results suggest that the detection of circulating miR-BART17-5p after treatment is a candidate biomarker at least comparable to those of BamHI-W DNA to predict treatment outcomes, and, thus, to select patients for adjuvant chemotherapy. In the previous two reports, patients with recurrence maintained high post-treatment EBV-DNA-positive rates of 90 and 63%, respectively [24,25]. In the current study, the EBV-DNA-positive rate in patients with relapse or residual disease at three months after treatment was 45% (5 out of 11), which was lower compared with these reports. In a review of the literature on the qPCR protocol to measure copy numbers of circulating EBV-DNA, Yip et al. showed that plasma gives a higher sensitivity than serum in the optimal experimental protocol, although the specificity remains the same [14]. If we had used plasma instead of serum after treatment, a higher sensitivity of EBV-DNA might have been obtained. For miR-BART17-5p, Gourzones et al. detected circulating miRNA in 10 out of 10 control donors using plasma samples and qPCR with 45 cycle [26]. In contrast, miR-BART17-5p was detected in 2 out of 40 control donors using serum and qPCR with 40 cycles in our current study. It is a problem that there is not a common protocol to measure these circulating DNAs and RNAs. The protocols should be established and unified to an optimal protocol in the future to compare these controversial results derived from different protocols.

Circulating miRNAs in malignant diseases are being actively investigated, and large amounts of data have already been published [26,27,28]. One remarkable characteristic of circulating miRNAs is their stability. To a large extent, the stability of circulating miRNAs results from their associations with various types of carriers [29]. BART-miRNAs packaged into exosomes have sufficient stability and motility to reach the circulating blood [22,29,30]. Interestingly, circulating miR-BART17-5p is co-purified with a protein-rich fraction but not with exosomes, which suggests the non-exosomal transport of miR-BART17-5p [26]. Arroyo et al. found that the majority of circulating miRNAs were cofractionated with protein complexes rather than with vesicles, and the majority of miRNAs studied were copurified with the Argonaute (AGO) 2 ribonucleoprotein complex [31]. In addition, AGO2 over-expression is a risk factor for advanced lymph node metastasis, and it can contribute to malignant behavior in NPC [32]. Therefore, it would be interesting to examine whether miR-BART17-5p can be immunoprecipitated with anti-AGO2 antibodies.

At least in 4 of the 11 recurrent or residual cases, the copy numbers of circulating miR-BART17-5p were increased from undetectable to detectable levels after treatment (S1 Table. Case 2, 3, 6, and 8). Although the mechanism of increase in miR-BART17-5p remains to be clarified in a future study, the changes in expression patterns of miRNAs by cancer treatment modalities such as radiotherapy and/or chemotherapy are being increasingly recognized. It has been shown in cancer cells that the expression of miRNAs may vary depending on parameters like the cell type, post-radiation time, and radiation dose [33,34,35]. The changes in expression profiles of miRNAs might also be observed in NPC after radiotherapy and/or chemotherapy. As miR-BART17-5p is released by an exosome-independent mechanism, the increase of circulating miR-BART17-5p might indicate a change in the secretory phenotype of NPC cells from exosome-dependent to exosome-independent after chemoradiotherapy. These are compatible with the hypotheses of Arroyo et al., in which vesicle-associated versus AGO2 complex-associated miRNAs originate from different cell types and reflect a cell-type-specific miRNA expression and/or release mechanism [31].

It has been shown that miR-BART17-5p can target the 3’UTR of the *LMP1* gene and negatively regulate the appropriate expression of the protein. miR-BART17-5p also modulates the LMP1-induced NF-κB signal transduction pathway and alleviates the cisplatin sensitivity of LMP1-expressing NPC cells [36]. High-level LMP1 inhibits cell growth and causes apoptosis, while low-level LMP1 promotes the growth and survival of these cells [20]. Indeed, miR-BART17-5p may devote a function of LMP1 to the cancer promotion side by targeting LMP1. In addition, miR-BART17-5p activates the Wnt pathway by down-regulating Wnt inhibitory genes, APC [21,37,38]. These data suggest that miR-BART17-5p helps transform the proapoptotic phenotype of LMP1-positive NPC cells to a surviving and chemo-resistant phenotype. The role of miR-BART17-5p in surviving NPC cells after chemotherapy and/or radiotherapy should be clarified in the future.

Some reports showed that non-EBV-related miRNAs may be biomarkers for the diagnosis of and prognosis associated with NPC [39,40,41]. Although these miRNAs were useful biomarkers at the initial diagnosis, it is unknown whether they are reliable biomarkers predicting recurrence and/or residual tumors after treatment. For EBV-related miRNAs, Zhang et al. reported that miR-BART7 and miR-BART13 are biomarkers not only for NPC diagnosis, but also for estimating the effect of therapy after treatment [15]. Although they showed that these EBV-related miRNAs are significantly decreased after treatment, they did not assess the correlation between the copy numbers of these miRNAs after treatment and the prognosis of each patient. Although our current report presents data from a pilot study, this is the first report to propose the clinical significance of quantifying copy numbers of EBV-specific miRNAs to estimate residual tumor cells after treatment in NPC patients.

In summary, circulating BamHI-W DNA was a useful biomarker for the diagnosis of NPC before treatment. The detection of circulating miR-BART17-5p after treatment was considered as a candidate potential biomarker to predict a poor treatment outcome. In the current study, the case number was too limited to arrive at a definite conclusion. However, the data shown in this study are encouraging to test whether the quantitative analysis of circulating miR-BART17-5p, an exosome-independent secretory EBV-associated miRNA, is a reliable potential biomarker to predict the treatment outcome with larger sample sizes in future studies.

## Supporting Information

S1 Table Copy numbers of circulating BamHI-W DNA, miR-BART2-5p, miR-BART17-5p, and miR-BART18-5p before and after treatment. (XLS)

S2 Table Copy numbers of circulating BamHI-W DNA, miR-BART2-5p, miR-BART17-5p, and miR-BART18-5p in non-NPC controls. (XLS)

S3 Table Detection of circulating EBV-DNA or EBV-RNA in patients with nasopharyngeal carcinoma. (XLS)
